# Supplementary material for: Surface-confined alternating copolymerization with molecular precision by stoichiometric control
Source: Nat Commun. 2024 Jan 22;15:666. doi: 10.1038/s41467-024-44955-3 (PMC10803352; doi:10.1038/s41467-024-44955-3)
Supplement: Supplementary file 1 — Supplementary Information [file 41467_2024_44955_MOESM1_ESM.pdf]

Supplementary Information for

Surface-Confined Alternating Copolymerization with Molecular Precision by Stoichiometric Control

Lingbo Xing,[1,]† Jie Li,[2,]† Yuchen Bai, [1,]† Yuxuan Lin,[1] Lianghong Xiao,[1] Changlin Li,[1] Dahui Zhao,[1] Yongfeng Wang,[2,] Qiwei Chen,[1,]* Jing Liu,[1,]* Kai Wu[1,]**

[1]BNLMS, College of Chemistry and Molecular Engineering, Peking University, Beijing 100871, China

[2]Center for Carbon-based Electronics and Key Laboratory for the Physics and Chemistry of Nanodevices, School of Electronics, Peking University, Beijing 100871, China.

Emails: kaiwu@pku.edu.cn, jing.liu@pku.edu.cn, chenqw@pku.edu.cn, yongfengwang@pku.edu.cn.

†These authors contributed equally.

Supplementary Figures

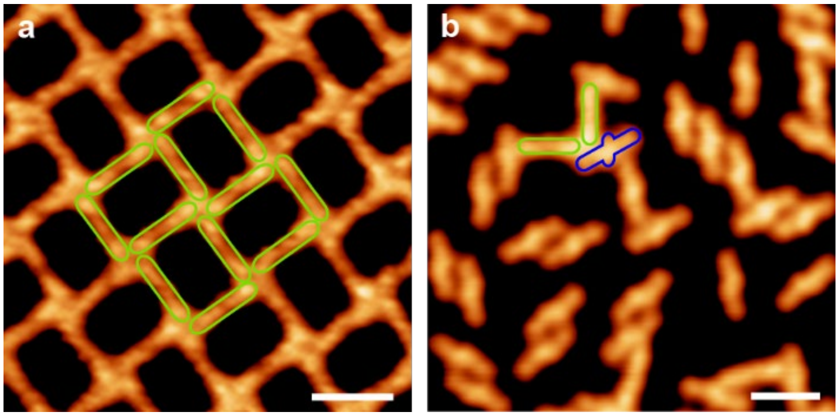

Supplementary Fig. 1 STM images of (a) a self-assembled structure of P-Br (Scanning conditions: bias voltage *V* = 10 mV and tunneling current *I* = 100 pA), and (b) randomly absorbed A-H and P-Br (*V* = 10 mV and *I* = 100 pA). The A-H and P-Br monomers are highlighted by the dark blue and green frames, respectively. Scale bars: 2 nm.

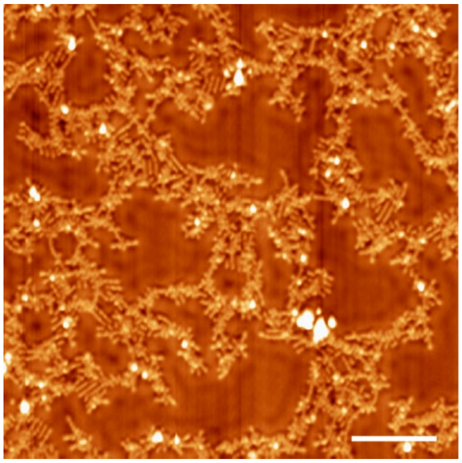

Supplementary Fig. 2 STM image of the less ordered covalent products obtained by thermal annealing of the A-H + P-Br sample at ~ 410 K (*V* = 100 mV and *I* = 100 pA). Scale bar: 15 nm.

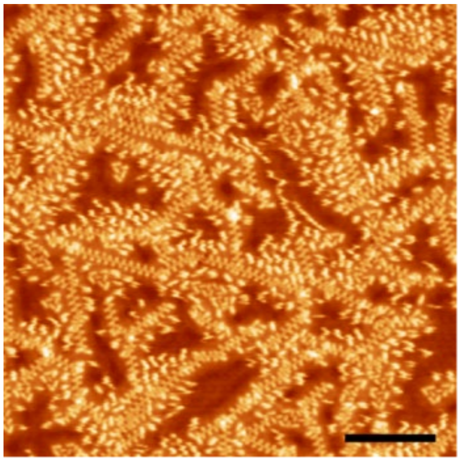

Supplementary Fig. 3 STM image showing the fuzzy topograph of the P-H molecules due to their high mobility (V = 100 mV and I = 100 pA). Scale bar: 16 nm.

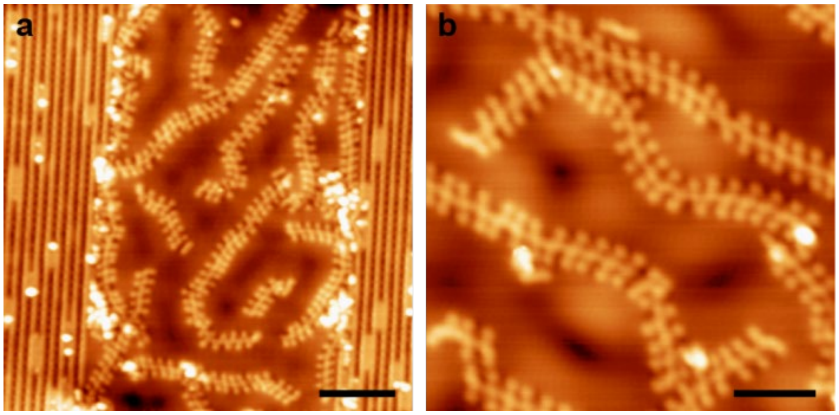

Supplementary Fig. 4 (a) STM image of the $[P]_n$ + A-H sample after its thermal treatment at ~ 350 K (V = 100 mV and I = 30 pA). (b) Magnified STM image of the molecular chains formed by covalent C-C coupling of A-H after thermal treatment of the sample at ~ 350 K (V = 10 mV and I = 30 pA). Scale bars: (a) 8 nm, (b) 4 nm.

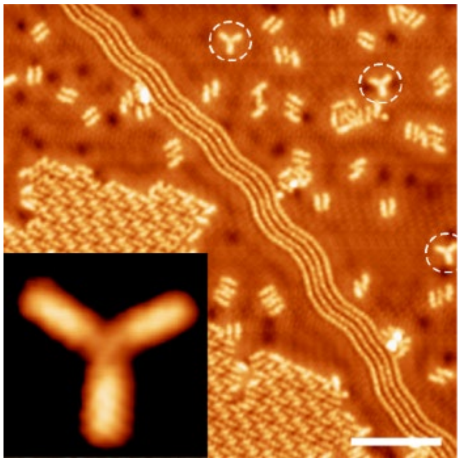

Supplementary Fig. 5 STM image of the [P-Ag]$_n$ + A-H sample at the early reaction stage (V = 100 mV and I = 100 pA). The three-lobed molecular structures, as highlighted by the white dashed circles, are ascribed to the trimers of the phenyl residues with their debrominated sites anchored to the substrate Ag atoms[1]. Inset: High-resolution STM image of a phenyl residue trimer (V = 10 mV and I = 100 pA). Scale bar: 12 nm.

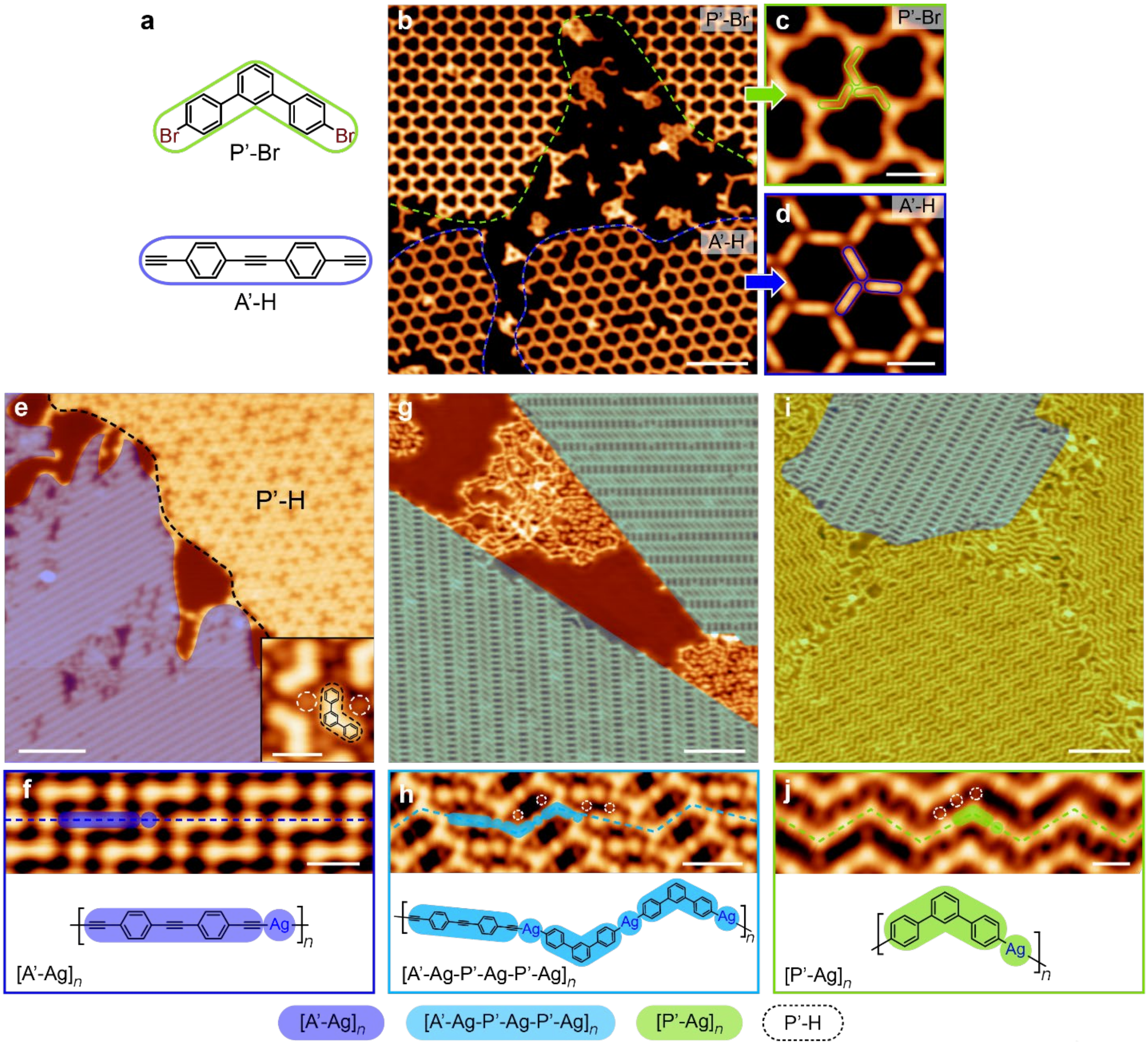

Supplementary Fig. 6 Surface-confined reaction between P'-Br and A'-H. (a) Chemical structures of P'-Br and A'-H. (b) STM image of the co-existent self-assembly domains of P'-Br and A'-H (V = 1 V and I = 100 pA). Scale bar: 10 nm. High-resolution STM images of the self-assemblies of (c) P'-Br (V = 300 mV and I = 200 pA) and (d) A'-H (V = 300 mV and I = 200 pA). Scale bars: 2 nm. Large-area STM images of the reaction products achieved by P'-Br and A'-H on Ag(111) at (e) $r \sim 0.7$ (V = 1 V and I = 100 pA), (g) $r \sim 3$ (V = 300 mV and I = 200 pA), and (i) $r \sim 8$ (V = 300 mV and I = 200 pA). Scale bars: 10 nm. Inset of (e): High-resolution STM image of the co-assembly formed by P'-H and Br adatoms (V = 300 mV and I = 200 pA). Scale bar: 1 nm. High-resolution STM images of (f) the [A'-Ag]$_n$ chains (V = 100 mV and I = 200 pA), (h) the [A'-Ag-P'-Ag-P'-Ag]$_n$ chains (V = 600 mV and I = 200 pA), and (j) the [P'-Ag]$_n$ chains (V = 300 mV

and I = 200 pA). The extending orientations of the chains are highlighted by the colored dashed lines. The chemical structures of the polymeric products are illustrated in bottom panels of (f), (h) and (j). Scale bars: 1 nm for (f) and (j), and 2 nm for (h).

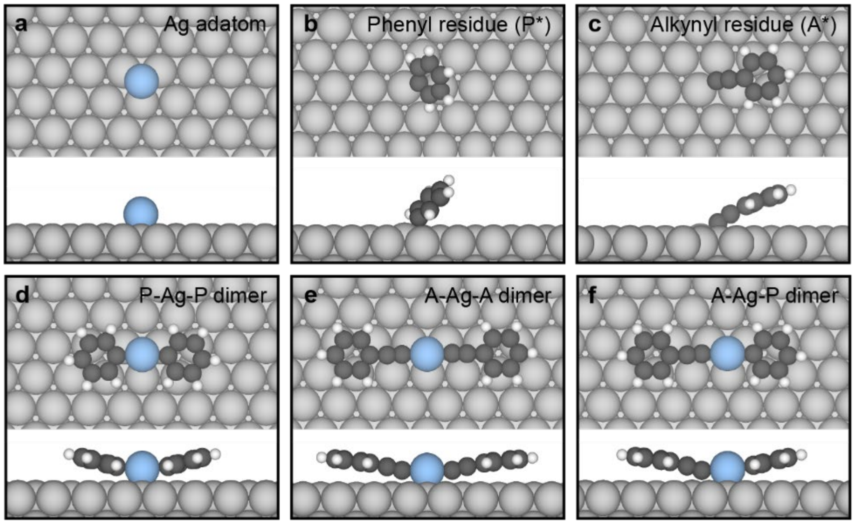

Supplementary Fig. 7 Optimized models of (a) a Ag adatom, (b) a phenyl residue, (c) a alkynyl residue, (d) a P-Ag-P dimer, (e) an A-Ag-A dimer, and (f) an A-Ag-P dimer adsorbed on Ag(111). Color code: white for H, dark gray for C, light gray for substrate Ag, and light blue for Ag adatom.

Supplementary Tables

Supplementary Table 1. Overall Reactions at Varied *r* Intervals.

Reactants	r	Overall Reaction
A-H + P-Br	$0 < r \leq 1$	n A-H + n P-Br + n Ag → $[\text{A-Ag}]_n$ + n Br + n P-H
	$1 < r \leq 2$	n A-H + rn P-Br + rn Ag → (r-1) $[\text{A-Ag-P-Ag}]_n$ + (2-r) $[\text{A-Ag}]_n$ + rn Br + n P-H
	$r > 2$	n A-H + rn P-Br + rn Ag → $[\text{A-Ag-P-Ag}]_n$ + (r-2) $[\text{P-Ag}]_n$ + rn Br + n P-H
$[\text{A-Ag}]_n$ + P-Br	$1 < r \leq 2$	$[\text{A-Ag}]_n$ + n P-Br + n Ag → $[\text{A-Ag-P-Ag}]_n$ + n Br
	$r > 2$	$[\text{A-Ag}]_n$ + (r-1)n P-Br + (r-1)n Ag → $[\text{A-Ag-P-Ag}]_n$ + (r-2) $[\text{P-Ag}]_n$ + (r-1)n Br
$[\text{P-Ag}]_n$ + A-H	$0 < r \leq 1$	$[\text{P-Ag}]_n$ + n A-H → $[\text{A-Ag}]_n$ + n P-H
	$1 < r \leq 2$	r $[\text{P-Ag}]_n$ + n A-H → (r-1) $[\text{A-Ag-P-Ag}]_n$ + (2-r) $[\text{A-Ag}]_n$ + n P-H
	$r > 2$	2 $[\text{P-Ag}]_n$ + n A-H → $[\text{A-Ag-P-Ag}]_n$ + n P-H

r and *n* reffer to the P-Br : A-H ratio and the number of repeating units in the polymers, respectively.

Supplementary Table 2. Formation Energies for the Organometallic Nodes.

Structure	Formation Energy (eV)
A-Ag-A	-0.940
P-Ag-P	-1.299
A-Ag-P	-1.144

Supplementary Discussion

1. Determination of Stoichiometric Ratios

The stoichiometric ratio between the P-Br and A-H monomers was experimentally controlled by their evaporation times at specific evaporation fluxes, and then confirmed by exhausting counting of both monomer numbers on the sampled surface. The STM images used for the statistical analyses are acquired at random spots of the substrate surface. The ratio value, *r*, is calculated by the weighted average of the P-Br molecular density in the assembled and disordered structures on the surface divided by that of A-H. The calculated molecular density of an ordered molecular assembly is the number of the molecules within a unit cell divided by the unit cell area. The molecular density of a less ordered zone is estimated by the total number of the molecules divided by the zone area that is experimentally monitored.

2. Estimation of $E_\mathrm{r}^\mathrm{homo}$ and E_r^co

$E_\mathrm{r}^\mathrm{homo}$ and E_r^co refer to the reaction energies per A* involved of the polymerization Reactions (1) and (2), respectively, as listed below:

Reaction (1): n A* + n P* + 2n Ag → $[\text{A-Ag}]_n$ + $[\text{P-Ag}]_n$,

and Reaction (2): n A* + n P* + 2n Ag → $[\text{A-Ag-P-Ag}]_n$.

The reaction energy for Reaction (1) can be estimated by summation of the formation energies for one P-Ag-P ($E_\mathrm{f}^\mathrm{P-Ag-P}$) and one A-Ag-A ($E_\mathrm{f}^\mathrm{A-Ag-A}$) intermolecular organometallic connections,

$$E_\mathrm{r}^\mathrm{homo} = E_\mathrm{f}^\mathrm{P-Ag-P} + E_\mathrm{f}^\mathrm{A-Ag-A}$$

$$= E_\mathrm{P-Ag-P/Ag(111)} + E_\mathrm{A-Ag-A/Ag(111)} - 2E_\mathrm{P^*/Ag(111)} - 2E_\mathrm{A^*/Ag(111)} - 2E_\mathrm{Ag/Ag(111)} + 4E_\mathrm{Ag(111)}, \quad (1)$$

while that for Reaction (2), twice the formation energy for one A-Ag-P node ($E_\mathrm{f}^\mathrm{A-Ag-P}$),

$$E_\mathrm{r}^\mathrm{co} = 2E_\mathrm{f}^\mathrm{A-Ag-P} = 2(E_\mathrm{A-Ag-P/Ag(111)} - E_\mathrm{P^*/Ag(111)} - E_\mathrm{A^*/Ag(111)} - E_\mathrm{Ag/Ag(111)} + 2E_\mathrm{Ag(111)}). \quad (2)$$

$E_\mathrm{P-Ag-P/Ag(111)}$, $E_\mathrm{A-Ag-A/Ag(111)}$ and $E_\mathrm{A-Ag-P/Ag(111)}$ refer to the energies of the P-Ag-P (Supplementary Fig. 7d), A-Ag-A (Supplementary Fig. 7e) and A-Ag-P (Supplementary Fig. 7f) dimers adsorbed on Ag(111), correspondingly. $E_\mathrm{Ag/Ag(111)}$, $E_\mathrm{P^*/Ag(111)}$ and $E_\mathrm{A^*/Ag(111)}$ correspond to the energy of the a Ag adatom (Supplementary Fig. 7a), a P* residue (Supplementary Fig. 7b) and an A* residue (Supplementary Fig. 7c) on Ag(111). $E_\mathrm{Ag(111)}$ is the energy of the Ag(111) substrate. These energies are calculated based on the simplified models shown in Supplementary Fig. 7. The accordingly

achieved formation energies for the organometallic structures are listed in Supplementary Table 2. As a result, the reaction energies are $E_\mathrm{r}^\mathrm{homo}$ = -2.24 eV and E_r^co = -2.29 eV.

3. Estimation of Product Yields

The yields of the [A-Ag]*n* ($Y_{[\mathrm{A-Ag}]_n}$), [A-Ag-P-Ag]*n* ($Y_{[\mathrm{A-Ag-P-Ag}]_n}$) and [P-Ag]*n* ($Y_{[\mathrm{P-Ag}]_n}$) products are defined as the proportions of the intermolecular A-Ag-A, A-Ag-P and P-Ag-P connections to the total amount of the organometallic nodes in the system, correspondingly. To estimate the product yields of a specific sample, the STM images at random locations of the sample are first acquired (total scanning area ≥ 32000 nm^2). Then, the molecular densities of the A-Ag-A ($d_{\mathrm{A-Ag-A}}$), A-Ag-P ($d_{\mathrm{A-Ag-P}}$), and P-Ag-P ($d_{\mathrm{P-Ag-P}}$) nodes in assembled or less ordered structures are estimated. The molecular density in the ordered molecular assemblies is calculated by dividing the number of the organometallic nodes in a unit cell by the area of the unit cell. The molecular density in the less-ordered regions is estimated by dividing the total number of the organometallic nodes by the total area of the less ordered regions that are experimentally inspected (≥ 2000 nm^2). The coverage proportion (C) of each ordered or less ordered structure is estimated by dividing the area of the specific structure by the total area that is experimentally inspected. The overall molecular densities of the organometallic nodes ($D_{\mathrm{A-Ag-A}}$, $D_{\mathrm{A-Ag-P}}$ and $D_{\mathrm{P-Ag-P}}$) are correspondingly estimated as:

$$D_{\mathrm{A-Ag-A}} = \sum_i C_i d_{\mathrm{A-Ag-A}_i}, \quad (3)$$

$$D_{\mathrm{A-Ag-P}} = \sum_i C_i d_{\mathrm{A-Ag-P}_i}, \quad (4)$$

$$\text{and } D_{\mathrm{P-Ag-P}} = \sum_i C_i d_{\mathrm{P-Ag-P}_i}. \quad (5)$$

$Y_{[\mathrm{A-Ag}]_n}$, $Y_{[\mathrm{A-Ag-P-Ag}]_n}$ and $Y_{[\mathrm{P-Ag}]_n}$ are therefore calculated as:

$$Y_{[\mathrm{A-Ag}]_n} = D_{\mathrm{A-Ag-A}}/(D_{\mathrm{A-Ag-A}} + D_{\mathrm{A-Ag-P}} + D_{\mathrm{P-Ag-P}}), \quad (6)$$

$$Y_{[\mathrm{A-Ag-P-Ag}]_n} = D_{\mathrm{A-Ag-P}}/(D_{\mathrm{A-Ag-A}} + D_{\mathrm{A-Ag-P}} + D_{\mathrm{P-Ag-P}}), \quad (7)$$

$$\text{and } Y_{[\mathrm{P-Ag}]_n} = D_{\mathrm{P-Ag-P}}/(D_{\mathrm{A-Ag-A}} + D_{\mathrm{A-Ag-P}} + D_{\mathrm{P-Ag-P}}). \quad (8)$$

4. Reaction of 1,2-Bis(4-ethynylphenyl)ethyne and 4,4''-Dibromo-1,1':3',1''-terphenyl on Ag(111)

Co-evaporation of 4,4''-dibromo-1,1':3',1''-terphenyl (upper in Supplementary Fig. 6a, denoted as P'-Br) and 1,2-bis(4-ethynylphenyl)ethyne (lower in Supplementary Fig. 6a, denoted as A'-H) onto the Ag(111) substrate held below RT led to their self-assemblies into separate domains, as shown in Supplementary Fig. 6b-d.

An *r*-dependent (r = P'-Br : A'-H) homo-/co-polymerization selectivity was noticed in the A'-H + P'-Br system after annealing of the samples pre-covered by the two monomers at varied stoichiometric ratios at RT - 340 K. On the samples at $r \leq 1$ (e.g., $r \sim 0.7$, Supplementary Fig. 6e), the homopolymeric [A'-Ag]$_n$ chains (shaded in dark blue) appeared as the main product. The assembled [A'-Ag]$_n$ chains coexisted with less ordered domains (marked by the dashed black frame in Supplementary Fig. 6e) formed by 1,1':3',1''-terphenyl (denoted as P'-H, highlighted by the dashed black frame in the inset) and the detached Br adatoms (marked by the dashed white circles). P'-H is presumably the byproduct due to the passivation of the debrominated P'-Br by the H atoms produced from the A-H dehydrogenation, according to our mechanistic analyses in the main text. A high-resolution STM image of the [A'-Ag]$_n$ chains is given in Supplementary Fig. 6f, showing the alternate arrangement of the rod-like molecular moieties and the circular protrusions assigned as the Ag adatoms.

Once P'-Br is in far excess to A'-H (e.g., $r \sim 8$, Supplementary Fig. 6i), the surface of the annealed sample was dominantly covered by the zigzag chains (shaded in green) that were identified as the [P'-Ag]$_n$ homopolymers according to previous reports[2,3]. A closeup of the zigzag chains (Supplementary Fig. 6j) resolves the alternate arrangement of the 120° V-shaped molecular moieties and the Ag adatoms, i.e., the round and bright protrusions. Detached Br adatoms appear as the dim dots located in between the [P'-Ag]$_n$ chains, as marked by the dashed white circles in Supplementary Fig. 6j. In addition to the main product [P'-Ag]$_n$, the copolymeric structures (discussed below) were also obtained, as shaded in light blue in Supplementary Fig. 6i.

At middle sociometric ratios, copolymers with an ordered sequence (shaded in light blue) became the main product on the surface, in coexistence with the less ordered structures composed of randomly aligned organometallic chains and the P'-H molecules. The high-resolution STM image of the copolymers (Supplementary Fig. 6h) reveals a [A'-Ag-P'-Ag-P'-Ag]$_n$ sequence, indicating that the product is an "ABB" type copolymer. The yield of the [A'-Ag-P'-Ag-P'-Ag]$_n$ copolymer maximizes at $r \sim 3$ (Supplementary Fig. 6g). The dim dots located in between the

copolymeric chains, as marked by the dashed white circles in Supplementary Fig. 6h, are the detached Br adatoms.

The on-surface reaction between P'-Br and A'-H shows an r-dependent homo-/co-polymerization selectivity similar to that between P-Br and A-H. When the amount of the bromide is fewer than ($r \leq 1$) and far in excess to that of the alkyne, the homopolymers $[A\text{-}Ag]_n$ (or $[A'\text{-}Ag]_n$) and $[P\text{-}Ag]_n$ (or $[P'\text{-}Ag]_n$) become the main products, respectively. At middle ratios, the copolymers with ordered sequences prevail on the surface. These findings suggest the similarities in two aspects of the reaction mechanisms for both systems. One is the activation of the alkyne monomers consumes the bromides of equivalent amount following the reaction,

$$\text{A-H (or A'-H) + P-Br (or P'-Br)} \rightarrow \text{A* (or A'*) + P-H (or P'-H) + Br,}$$

which explains the selective homopolymerization of A-H (or A'-H) to give $[A\text{-}Ag]_n$ (or $[A'\text{-}Ag]_n$) at $r \leq 1$ and the appearance of the P-H (or P'-H) byproduct. The other is the preferred copolymerization rather than the homopolymerization once both A* (or A'*) and P* (or P'*) are available in the reaction systems.

In terms of the "ABB" sequence of the copolymeric product formed by A'-H and P'-Br, our speculation is twofold. On the one hand, the chain periodicity of the $[A'\text{-}Ag\text{-}P'\text{-}Ag\text{-}P'\text{-}Ag]_n$ copolymer is in commensuration with the substrate lattice along the orientations the chains extend. Such a chain-substrate commensurability may selectively stabilize the ABB-type copolymer. On the other hand, the formation of the well-ordered and close-packed assemblies of the $[A'\text{-}Ag\text{-}P'\text{-}Ag\text{-}P'\text{-}Ag]_n$ chains (Supplementary Fig. 6g and h) may also contribute to their stabilization.

Supplementary References

1. Zhou, X. et al. Steering surface reaction dynamics with self-assembly strategy. *Angew. Chem. Int. Ed.* **56**, 12852-12856 (2017).
2. Fan, Q. et al. Surface-assisted formation, assembly, and dynamics of planar organometallic macrocycles and zigzag shaped polymer chains with C-Cu-C bonds. *ACS Nano* **8**, 709-718 (2014).
3. Fan, Q. et al. Surface-assisted organic synthesis of hyperbenzene nanotroughs. *Angew. Chem. Int. Ed.* **52**, 4668-4672 (2013).
